# Elucidating the Impact of Payload Conjugation on the Cell-Penetrating Efficiency of the Endosomal Escape Peptide dfTAT: Implications for Future Designs for CPP-Based Delivery Systems

**DOI:** 10.1021/acs.bioconjchem.3c00369

**Published:** 2023-09-29

**Authors:** Joshua Diaz, Miles Pietsch, Marissa Davila, Gerardo Jaimes, Alexis Hudson, Jean-Philippe Pellois

**Affiliations:** † Department of Biochemistry and Biophysics, Texas A&M University, College Station, Texas 77843, United States; ‡ Department of Chemistry, Texas A&M University, College Station, Texas 77843, United States

## Abstract

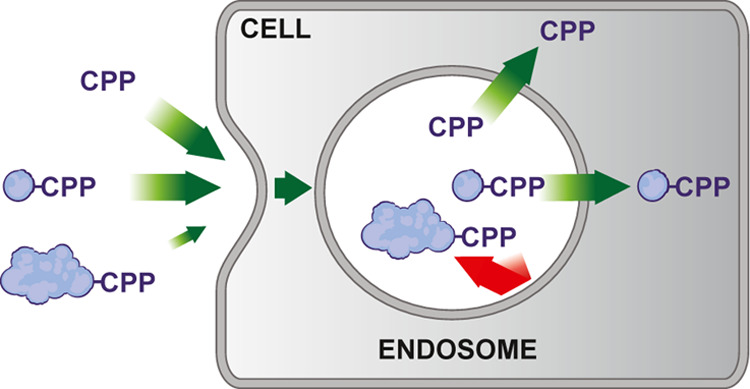

Cell-penetrating peptides (CPPs) are promising tools for the intracellular delivery of various biological payloads. However, the impact of payload conjugation on the cell-penetrating activity of CPPs is poorly understood. This study focused on dfTAT, a modified version of the HIV-TAT peptide with enhanced endosomal escape activity, to explore how different payloads affect its cell-penetrating activity. We systematically examined dfTAT conjugated with the SnoopTag/SnoopCatcher pair and found that while smaller payloads such as short peptides do not significantly impair dfTAT’s cell delivery activity, larger payloads markedly reduce both its endocytic uptake and endosomal escape efficiency. Our results highlight the role of the payload size and bulk in limiting CPP-mediated delivery. While further research is needed to understand the molecular underpinnings of these effects, our findings pave the way for developing more effective CPP-based delivery systems.

## Introduction

Cell-penetrating peptides (CPP) represent a family of peptides that can cross biological membranes and enter human cells. A prototypical CPP is TAT, a peptide derived from the HIV transactivator of transcription (HIV-TAT).^1−3^ TAT has been used in numerous conjugation forms with various payloads, enabling diverse applications such as studying biomolecules in the cellular environment, gene editing, and cellular reprogramming.^4−7^ However, CPP-mediated delivery often remains inefficient, necessitating a further understanding of their mechanism of action and enhancement of their cell penetration activity.

One significant issue with CPPs like TAT is the unpredictability of how conjugating TAT to a payload impacts its cell penetration activity and efficiency. This unpredictability arises from the highly variable molecular features of CPPs and cargos.^8^ Researchers must empirically determine how their cargo of interest impacts the performance of the CPP, a process that must be repeated for each new cargo.^9−12^ Moreover, the study of CPPs is complicated by their often inefficient cell penetration, particularly their lack of endosomal escape capability. This inefficiency makes the cell penetration process often barely detectable and prone to variability, complicating the establishment of structure–activity relationships.

To solve these problems, several laboratories, including ours, have focused on improving the delivery efficiency of TAT, particularly its ability to escape from endosomes.^13−16^ We have discovered that a dimeric analogue of TAT, dfTAT, can escape from late endosomes and enter the cytosol with high efficiency.^15^ Mechanistically, this escape activity involves the dfTAT-induced leaky fusion of late endosomal membranes.^17−19^ The multivesicular nature of late endosomes and the presence of the anionic lipid bis(monoacylglycero)phosphate (BMP) contribute to the specific activity of dfTAT toward these organelles.^17^ The high efficiency of dfTAT provides a valuable starting point for studying the impact of CPP or payload changes on cell penetration, enabling us to establish structure–activity relationships with relative ease and gain a more quantitative understanding of cell penetration. This discovery has led to the efficient delivery of various payloads including enzymes, transcription factors, small molecules, peptides, and nanoparticles. We have used dfTAT in a co-incubation format for tissue culture applications.^20^ This approach is convenient because it does not require modifications of the payload, which simply needs to be endocytosed by cells along with dfTAT. The concentration of the payload can also be titrated independently of that of dfTAT, leading to relative control of how many molecules of the payload enter the cell. We have recently extended this approach to the *in vivo* setting, using stereotactic cortical injections of mouse brains.^21^ The co-incubation format was exploited for the successful delivery of Cre recombinase into neurons and astrocytes. However, the tissue region of successful delivery is limited to the vicinity of the injection site. Hence, as they diffuse away from the injection site, dfTAT and payload take different routes and become endocytosed separately instead of together, yielding a failed delivery. In turn, it may be preferable to attach dfTAT to its payload in this type of application.

In this study, we aimed to probe how attachment to a payload impacts the cell penetration of dfTAT. In particular, we aimed to establish whether the CPP would retain its valuable endosomal escape activity when conjugated to peptides or proteins. To achieve this, we designed a panel of dfTAT-payload conjugates and systematically measured the conjugation effects on dfTAT’s cell penetration efficiency. Our data establish a size limit that is tolerable to the function of dfTAT, providing valuable insights for future designs of delivery systems.

## Results

### Design and Characterization of Model dfTAT Payloads

**Figure 1:**
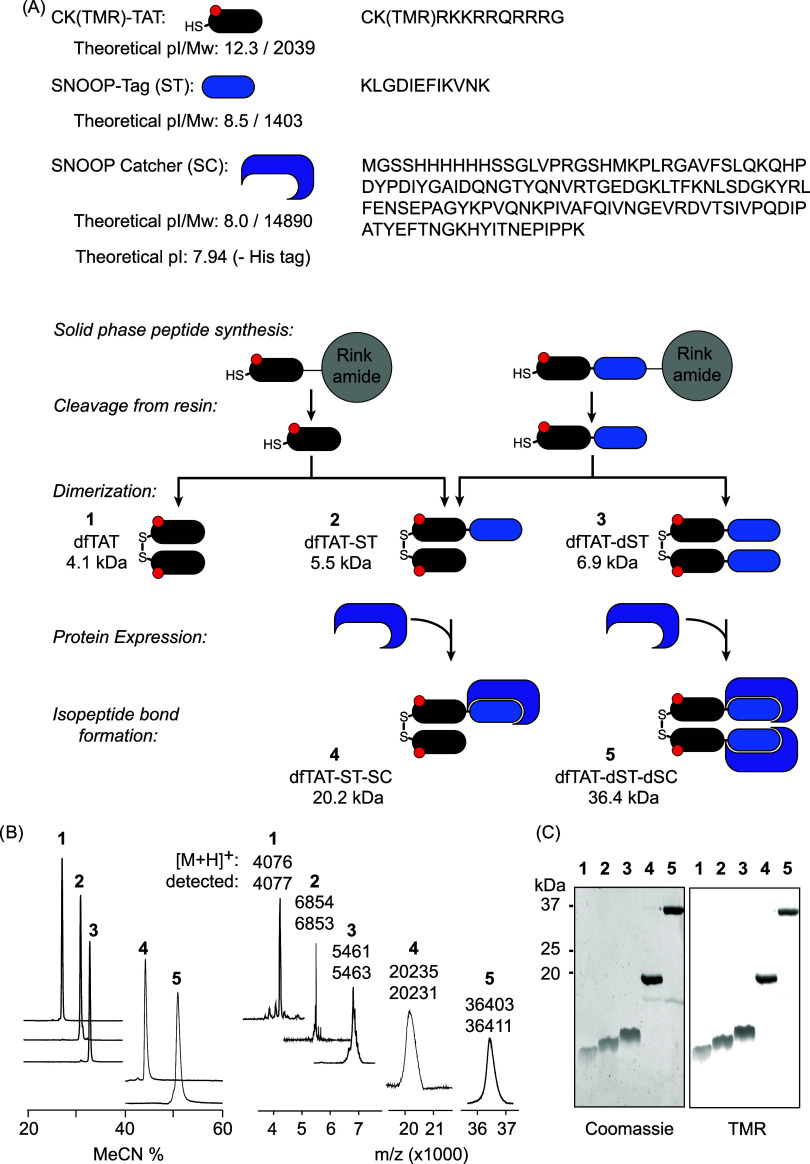
Characterization of dfTAT variants conjugated to SnoopTag and SnoopCatcher. (A) Peptide and protein modules used in this study and synthetic scheme used to assemble constructs **1** to **5**. (B) HPLC and mass spectrometry analyses of constructs. The *x*-axis for HPLC elution represents the percentage of acetonitrile in the mobile phase. (C) SDS-PAGE analysis of the constructs. The gel is imaged after Coomassie staining and by fluorescence emission of the TMR label.

dfTAT consists of two TAT peptides fluorescently labeled with tetramethylrhodamine (TMR) and linked by a disulfide bond (Figures 1A and S1). The dimerization of the arginine-rich peptide contributes to its high endosomal escape efficiency, the monomer analogue being relatively inactive.^15,22^ Notably, both monomer TAT and dfTAT follow similar routes of endocytic uptake.^17,23^ Hence, we reasoned that the results gathered from dfTAT would be in part applicable to TAT and other TAT-like CPPs. The fluorophores in dfTAT provide hydrophobicity also important for membrane translocation.^24^ In its current form, dfTAT is not genetically encodable. To fuse this CPP to model proteins, we chose a synthetic scheme that exploits the SnoopTag/SnoopCatcher (ST/SC) split domain.^25^ In this system, the reaction between ST and SC results in the formation of a covalent isopeptide bond and irreversible conjugation between the recombinant SC and the synthetic ST peptide (Figure 1A).^26^ Using this strategy, the ST peptide (GKLGDIEFIKVNKGY, 1.4 kDa) was installed on the C-terminus of fTAT (CK(TMR)RKKRRQRRR) via Solid Phase Peptide Synthesis. This fTAT-ST peptide (CKRKKRRQRRRGKLGDIEFIKVNK) was purified by high-performance liquid chromatography (HPLC) along with fTAT. The two peptides were mixed under oxidative conditions to promote disulfide bond formation and generation of products **1**, **2**, and **3** in one pot (Figure 1A). Each peptide was purified by HPLC. Peptides **2** and **3** were incubated with recombinantly expressed SC (14.8 kDa) to generate adducts with one or two copies of the protein, products **4** and **5**, respectively. The purity of the products (>98%) was established by HPLC, mass spectrometry, and sodium dodecyl sulfate polyacrylamide gel electrophoresis (SDS-PAGE) analyses (Figure 1B,C) Overall, the products span molecular weights ranging from 4.1 to 36.4 kDa. Aliquots were stored at −80 °C and thawed for individual experiments.

A caveat of the presented protein design relates to the presence of disulfide bonds. Specifically, disulfide bond shuffling could lead to **2** generating **1** and **3**, and **4** generating **1** and **5**. Samples were routinely analyzed and found to be pure and stable in storage for the duration of the project (also ruling out disulfide bond reduction). Nonetheless, one can envision that **2** and **4** undergo shuffling when exposed to cells (thereby generating dfTAT or **1**, which was already demonstrated to be cell-penetrating). Below we report on the activity of all constructs but focus on **3** and **5** given that these molecules are unable to generate **1**.

### Conjugation of SnoopCatcher to dfTAT Diminishes Cell Penetration

**Figure 2:**
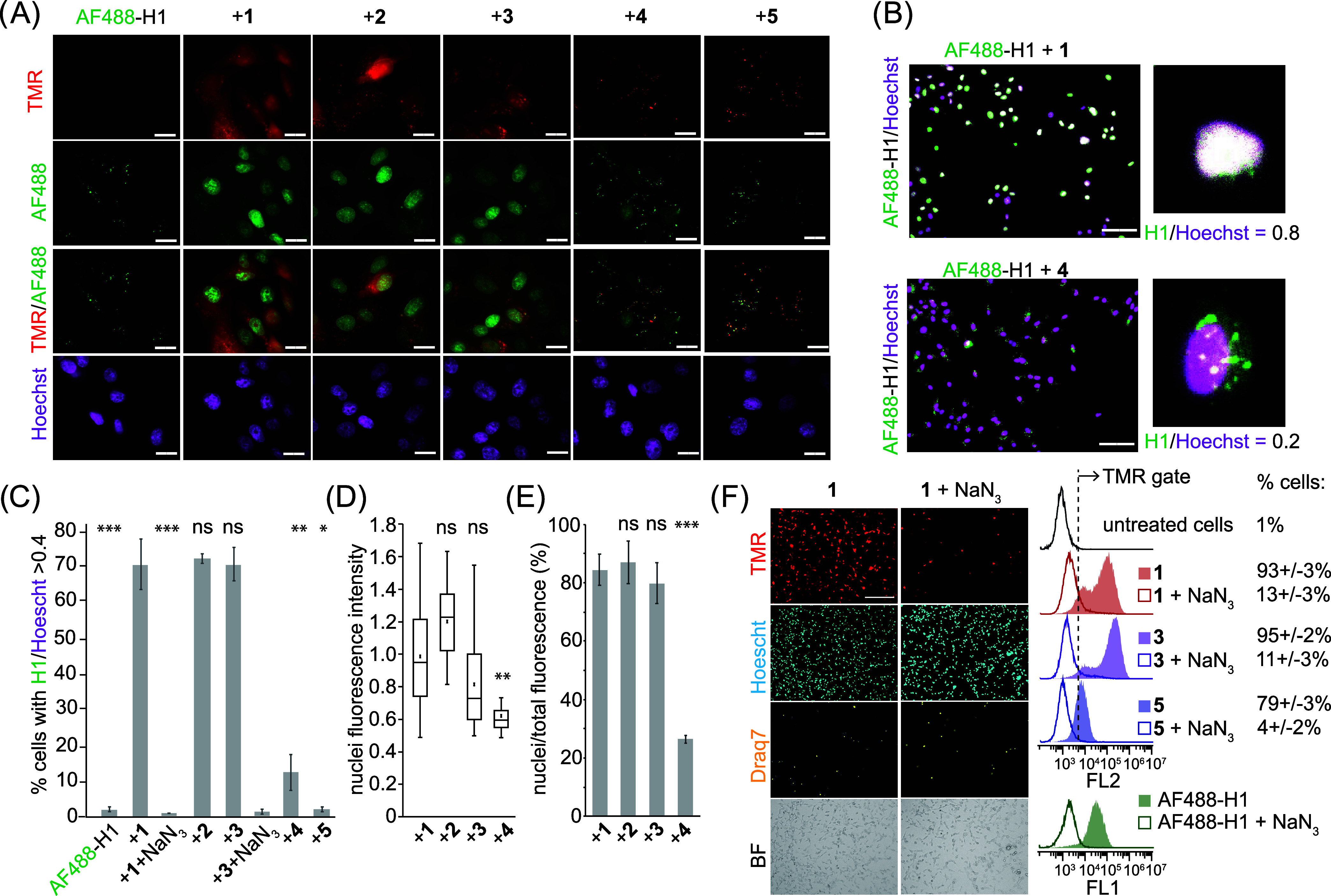
Cell penetration activity of constructs 1–5 as detected by the nuclear delivery of fluorescent histone AF488-H1. (A) Representative 100× fluorescence microscopy images of 1–5 (5 μM) co-incubated with AF488-H1 (1.25 μM) for 1 h (scale bar = 10 μm). Images are pseudo-colored red for TMR, green for AF488, and magenta for the nuclear stain Hoechst. (B) Sample images from colocalization analysis of AF488-H1 and Hoechst (scale bar = 100 μm). The overlay between AF488-H1 (pseudo-colored green) and Hoechst (pseudo-colored magenta) is colored white. A zoom-in on a single nucleus illustrates high colocalization and high H1 nuclear delivery, with a colocalization intersection (shown as H1/Hoechst ratio) of 0.8 (co-incubation of AF488-H1 and **1**). In contrast, a zoom-in from co-incubation of AF488-H1 and **4** illustrates a colocalization intersection of 0.2 (endosomal puncta being at the periphery of the nucleus, in an out-of-focus). (C) Quantification of the percentage of cells displaying an H1/Hoechst colocalization greater than 0.4 after incubation between AF488-H1 and **1**–**5**. Conditions including sodium azide controls are also included. (D) Box and whisker plots of the fluorescence intensities of the nuclear signal of AF488-H1 after delivery with **1**–**4**. The data are normalized to the mean intensity obtained after the delivery with **1**. (E) Ratio of the AF488-H1 fluorescence signal in nuclei versus the whole cell for different incubation conditions. (F) Flow cytometry analysis of **1**, **3**, and **5** (5 μM, detected in channel FL2) and AF488-H1 (1.25 μM, detected in channel FL1) with and without sodium azide. Representative 10× fluorescence microscopy images show cells incubated with **1** in the absence or presence of sodium azide (scale bar = 300 μm). Draq7, pseudo-colored orange, is used to detect dead cells from microscopy and flow cytometry. For each condition, the percentage of cells exhibiting a TMR signal is indicated. This percentage is determined by flow cytometry using a fluorescence FL2 intensity gate, set such that 99% of untreated cells fall below this level. In (C–F), the data represented are the averages and corresponding SDs of biological *N* = 3 replicates, using 500 cells/condition for analysis. *p ≤ 0.05, **p ≤ 0.01, ***p ≤ 0.001. ns is not significant, or *p* > 0.05.

The cell penetration of dfTAT and analogues can, in principle, be monitored by live cell fluorescence microscopy and quantified by exploiting the propensity of the peptide to stain nucleoli upon cell entry (this specific localization ensures that the signal detected is indeed intracellular and not simply an out-of-focus signal from the peptide bound to the outside of the cell). However, prior studies have established that partial proteolytic degradation occurs during transit within the endocytic pathway and upon cell entry.^27,28^ The TMR fluorophore is then released from dfTAT and contributes a diffuse signal that obscures the nucleolar staining. The fluorescence that remains trapped in the endosomes is also partially masked. Recently, to facilitate quantification, we have used AlexaFluor 488-labeled Histone H1 (AF488-H1) as a probe of endosomal escape.^21,24^ When co-incubated with dfTAT-like CPPs, AF488-H1 enters cells with minimal degradation and intrinsically accumulates in nuclei. The number of cells that display nuclear staining can then be counted (above a detection threshold) and a ratio of cytoplasmic/endosomal versus nuclear signal established.^21^ Molecules **1**, **2**, **3**, **4**, or **5** (5 μM) were co-incubated with AF488-H1 (1.25 μM) and administered to MDA-MB-231 cells (Figure 2A). Alone, AF488-H1 exhibits a punctate distribution consistent with endocytic uptake and endosomal entrapment. In contrast, co-incubation with molecules **1**, **2**, and **3** results in a high percentage of cells with a nuclear H1 signal (Figure 2C). The nuclear fluorescence intensities of AF488-H1 for these three conditions are comparable. This fluorescence intensity also represents approximately 80% of the total fluorescence signal in the cell (including the endosomes and cytoplasm). These results indicate that **1**, **2**, and **3** have similar delivery capabilities. Consistent with the idea that CPP and AF488-H1 enter cells together, the TMR signal of **1**, **2**, and **3** (or degradation fragments) is also intracellular, with partial nucleolar staining (not quantified). In contrast, co-incubation of AF488-H1 with **4**, leads to a reduced number of cells with detectable nuclear AF488-H1. Only 10% of cells contain a nuclear AF488-H1 signal above the set threshold. The detected nuclear AF488-H1 signal is markedly lower when compared to samples with **1**, **2**, and **3** (Figure 2A–E). Likewise, co-incubation of AF488-H1 with **5** fails at delivering AF488-H1 in nuclei at detectable levels. For both **4** and **5**, the TMR and AF488 fluorescence signals are predominantly punctate. To test the involvement of endocytic uptake in the delivery process, we preincubated cells with sodium azide (40 mM, 30 min preincubation). Sodium azide disrupts cellular processes requiring adenosine triphosphate (ATP), including endocytosis (Figure 2C,F).^29−31^ Sodium azide inhibited the uptake of AF488-H1, **1**, **3**, and **5** as detected by flow cytometry (Figure 2D). Inhibition of uptake is not complete for **1** and **3**, indicating that NaN3 may not block endocytosis fully in approximately 10% of the cells under the conditions tested. Alternatively, the peptides may partially enter these cells via another route, potentially direct plasma membrane translocation. Critically, sodium azide almost completely abolishes the nuclear delivery of AF488-H1 by either **1** or **3** (Figure 2C). Hence, this indicates that even if some of the peptides may translocate across the plasma membrane in a few cells, endocytic uptake of **1** and **3** is a necessary step for the cytosolic delivery of the AF488-H1 probe. Finally, control experiments were performed with the reduced and monomeric analogues of **1**, **3**, and **5** (Figure S2). These constructs were endocytosed based on microscopy colocalization with LysoTracker Green but were unable to deliver AF488-H1.

### SC Does Not Interfere with dfTAT in Trans

**Figure 3:**
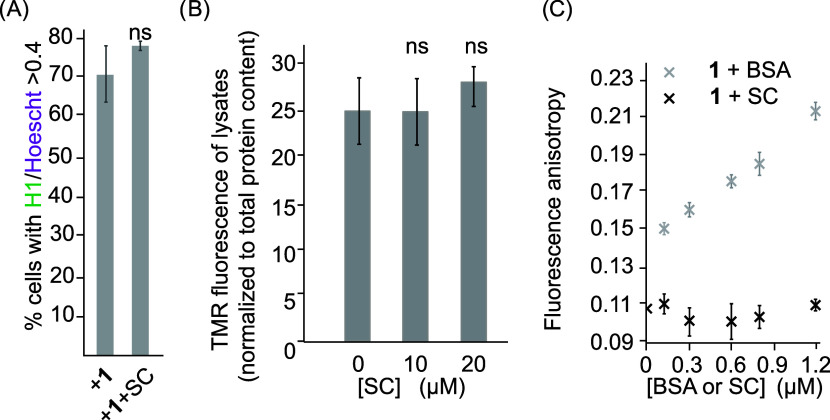
SC does not interfere with dfTAT when used in trans. (A) Quantification of the percentage of cells displaying an H1/Hoechst colocalization greater than 0.4 after incubation between AF488-H1 and **1 (**5 μM) with or without SC (10 μM). (B) Total uptake of **1** by cells in the absence or presence of SC. Total uptake is quantified by measuring the TMR signal associated with cells after lysis. This signal is normalized to the total protein content. (C) Fluorescence anisotropy of **1** titrated with increasing concentration of BSA (positive control) or SC. The data represented are the averages and corresponding SDs of biological *N* = 3 replicates for experiments with cells and technical triplicates for fluorescence anisotropy. Ns is not significant, or *p* > 0.05.

To understand why **5** loses cell penetration capability, we first tested whether SC inhibits dfTAT. SC (10 μM) was added to the AF448-H1 (1.25 μM) and **1** (5 μM) cocktail and incubated with cells as described in Figure 2. The percentages of cells with nuclear H1 were not statistically different between **1** and **1**+SC conditions (Figure 3A). The amount of **1** internalized by cells, quantified by measuring the total TMR signal of cell lysates, was unaffected by the presence of SC (Figure 3B). Moreover, the fluorescence anisotropy indicates that SC does not bind to **1** (Figure 3C). Collectively, these results suggest that SC does not interfere with dfTAT by interacting with the CPP directly or by competing with it for binding to cell components involved in uptake.

### dfTAT Conjugates **1** and **5** Colocalize with LysoTracker or AF488-H1 to a Similar Extent

**Figure 4:**
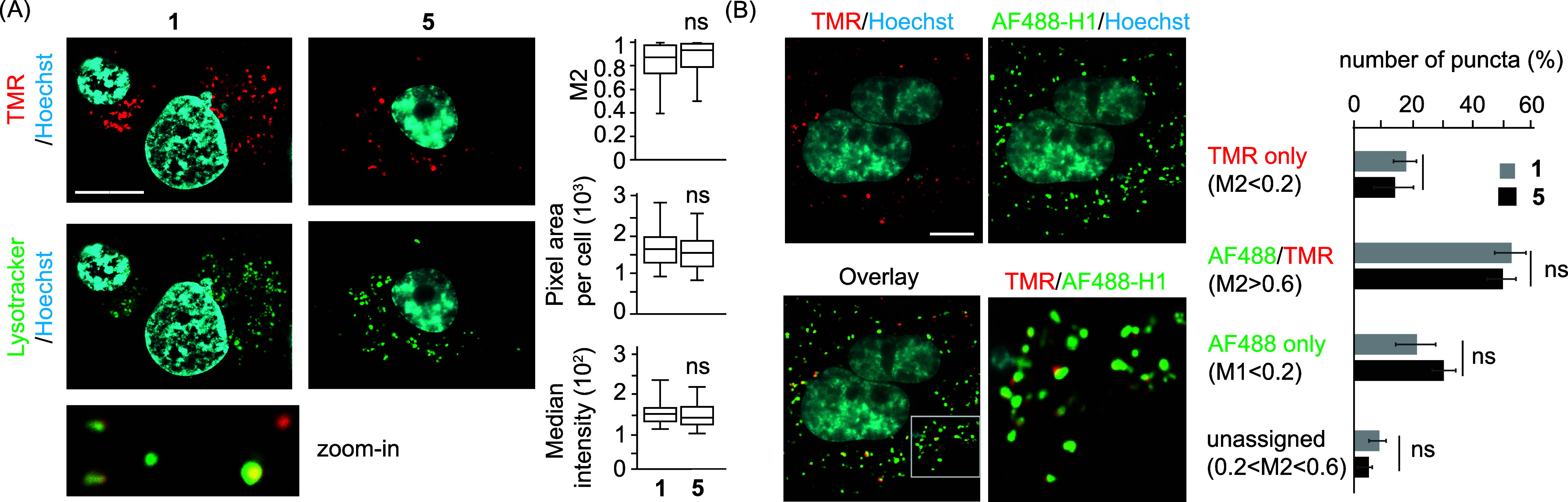
Distribution of **1** and **5** in endosomes. (A) Representative 100× fluorescence microscopy images of colocalization of **1** or **5** with LysoTracker Green (scale bar = 5 μm). The TMR fluorescence is pseudo-colored red, LysoTracker is green, and Hoechst is cyan. A zoomed-in view highlights overlay between green and red puncta as yellow. The Mander’s colocalization coefficient M2 (fraction of red signal that overlaps with green) is provided as a box and whisker plot. The total pixel area per cell that corresponds to fluorescent puncta and the median intensity of these pixels are shown. At least 500 cells were analyzed per replicate, with 3 biological replicates performed for each incubation conditions. (B) Representative 100× fluorescence microscopy images of colocalization of **5** with AF488-H1 (scale bar = 5 μm). The percentage of puncta that display a TMR signal only, an AF488 signal only, or both a TMR or AF488 signal are represented. Unassigned puncta are partially overlapping. The data are the averages and corresponding SDs of biological *N* = 3 replicates. Ns is not significant, or *p* > 0.05.

Prior reports have established that the colocalization of dfTAT and payload inside late endosomes is necessary for endosomal escape and release into the cytosol.^17,32^ Protein **5** could, therefore, fail to deliver AF488-H1 if its trafficking in the endocytic pathway is different from that of **1**, and if **5** does not colocalize with AF488-H1 within the endocytic pathway. To probe this possibility, we compared the colocalization of **5** and **1** with that of LysoTracker Green. LysoTracker Green stains acidic organelles, including late endosomes and lysosomes. Given that **1** escapes endosomes at 5 μM and that this prevents observation of endosomes, **1** was incubated at 0.5 μM for 1 h. Under these conditions, the fluorescence of **1** is exclusively punctate. The incubation of 1 μM probe **1** is equivalent to that of probe **5** at 5 μM (Figure 4A). Colocalization analysis between **1**/LysoTracker and **5**/LysoTracker was performed using Mander’s colocalization coefficients M1 and M2, giving the fraction of green fluorescence that overlaps with red and the fraction of red fluorescence that overlaps with green, respectively (Figure 4A). Based on this analysis, **1** and **5** colocalize with LysoTracker to a similar extent (mean M2 = 0.8 and 0.82, respectively, ∼80% red puncta are green). The pixel areas of TMR puncta, a proxy for the number of endosomes containing **1** or **5**, are similar between the two incubation conditions. The median intensities of these pixels are also indistinguishable under these conditions, indicating that **1** and **5** traffic through the endocytic pathway to reach late endosomes/lysosomes with similar propensities (note that these results are generally time-dependent and that longer wait times would likely lead to lysosomal accumulation).

In principle, constructs **1**–**5** must localize with AF488-H1 inside the same endosomes to mediate the endosomal escape of the histone into the cytosol. Therefore, we hypothesized that **5** might lose AF488-H1 delivery activity by simply taking a route different from that of AF488-H1 inside the endocytic pathway. To test for this hypothesis, we performed a colocalization analysis between **5**/AF488-H1 and compared it to that of **1**/AF488-H1. As described in the previous experiment, **5** was used at 5 μM while **1** was used at 0.5 μM to allow for comparison. Both constructs were incubated with AF488-H1 (1.25 μM) for 1 h, and cells were imaged using 100× microscopy (Figure 4B). Over 500 puncta were analyzed and binned into four categories: TMR only (M2 or fraction of red object that overlaps with green <0.2), AF488 only (M1 or fraction of green object that overlap with red), overlapping AF488/TMR puncta (M2 > 0.6), and unassigned (0.2 < M2 < 0.6). Based on this analysis, approximately 50% of the puncta containing **5** at a detectable level also contain AF488-H1. Notably, a similar result was obtained with **1**. This indicates that **5** and **1** have similar abilities to colocalize with the AF488-H1 payload.

### Conjugation to SnoopTag Does Not Impact Endosomal Uptake of dfTAT, but Conjugation to SnoopCatcher Does

**Figure 5:**
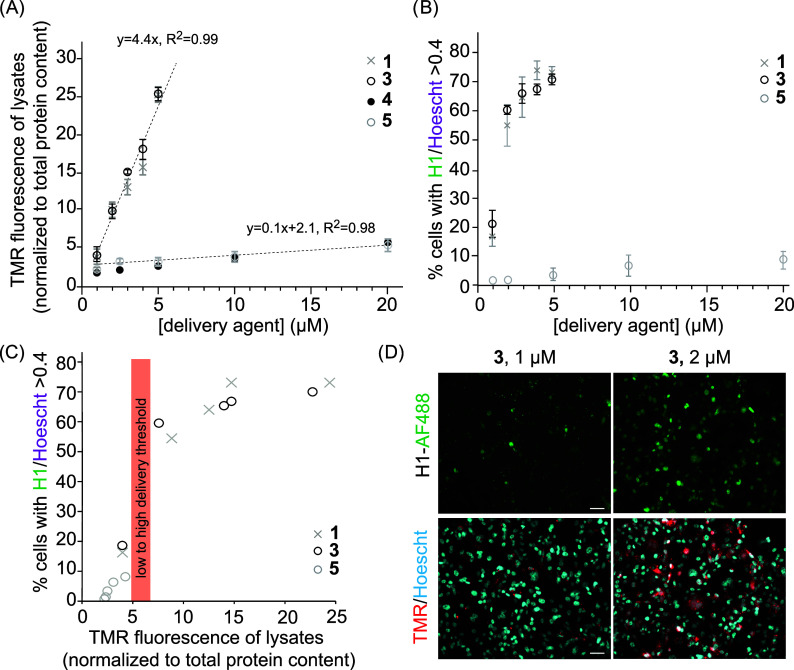
Attachment of SnoopCatcher to dfTAT hinders endocytic uptake. (A) Total uptake of **1**, **3**, **4**, and **5** as a function of extracellular concentration (1 h incubation). The data represented are the averages and corresponding SDs of biological *N* = 3 replicates. Linear curve fitting and the corresponding R2 for the 1–20 μM concentration range are provided. (B) Quantification of the percentage of cells displaying an H1/Hoechst colocalization intersection greater than 0.4 after incubation between AF488-H1 and **1**, **3**, or **5** as a function of the extracellular concentration of the delivery agents. The data represented are the averages and corresponding SDs of biological *N* = 3 replicates. (C) Plot representing the correlation between total uptake and H1 delivery efficiency of **1**, **3**, and **5**. These data combine results from parts (A) and (B). Standard deviations are omitted for clarity. An apparent uptake threshold at which the H1 delivery efficiency increases steeply is highlighted. (D) Representative 20× fluorescence images of cells incubated with **3** (1 or 2 μM) and AF488-H1 (scale bar = 50 μm).

Results from Figures 2 and 4 already indicated that MDA-MB-213 cells endocytose **1** and **3** at higher levels than **5**. Hence, **5** may fail to deliver AF488-H1 at 5 μM because this construct does not reach the endosomal concentration necessary to mediate membrane leakage and endosomal escape. To explore this scenario, we performed titration experiments in the 1–20 μM concentration range. For each condition, the amount of constructs **1**, **3**, and **5** internalized by cells was analyzed by measuring the total TMR fluorescence in cell lysates (Figure 5A). The nuclear delivery of AF488-H1 was quantified in parallel (Figure 5B). The total uptakes of **1** and **3** were similar, following a linear relationship with the concentration of constructs administered extracellularly (Figure 5A). At 1 μM, the uptake of **5** was approximately half that of **1** and **3**. In addition, the uptake increased only moderately at higher concentrations (Figure 5A). While the uptake of **1**/**3** increased 6-fold from 1 to 5 μM, the increase was only 1.9-fold for **5** in the 1 to 20 μM range. The relatively small increase in the uptake of **5** yielded a moderate increase in AF488-H1 delivery but with no more than 8% of cells displaying nuclear H1 at the maximum concentration tested (Figure 5B). In contrast, the delivery efficiency of **1** and **3** increases rapidly with concentration, jumping from approximately 20% at 1 μM to 60% at 2 μM (Figure 5B). Overall, these data establish that the delivery efficiency of **1** and **3** rapidly increases as an uptake threshold is reached (Figure 5C,D). In contrast, the uptake of **5** does not reach this threshold, and delivery efficiency remains poor.

### Conjugation of SnoopCatcher to dfTAT Inhibits Leakage of Lipid Bilayers

Achieving identical concentrations of **1**, **3**, and **5** inside endosomes would be ideal to evaluate the respective endosomal escape activity of these molecules precisely. However, the lower propensity of **5** for endocytic uptake prevents a direct comparison in live cells. To assess how **1**, **3**, and **5** interact with endosomal membranes, we therefore resorted to in vitro assays using large unilamellar vesicles (LUVs). These LUVs contain the lipids bis(monoacylglycero)phosphate (BMP), phosphatidylcholine (PC), and phosphatidylethanolamine (PE) at a ratio of 77:19:4. This composition mimics lipid bilayers present in late endosomes. dfTAT (**1**) induces the leaky fusion of this LUV model in a manner that is BMP-specific.^17,33,34^ Our prior results have indicated that the membrane disruption of 1 involves a two-step process: an initial fusion phase (with required contacts between lipid bilayers) followed by a leakage phase. Similar results have been reported with TAT.^19^ This LUV system recapitulates endosomal escape activity observed in cells with excellent fidelity, i.e., dfTAT analogues that escape endosomes cause LUV leakage while agents that stay trapped in endosomes do not.^17,33,34^

**Figure 6:**
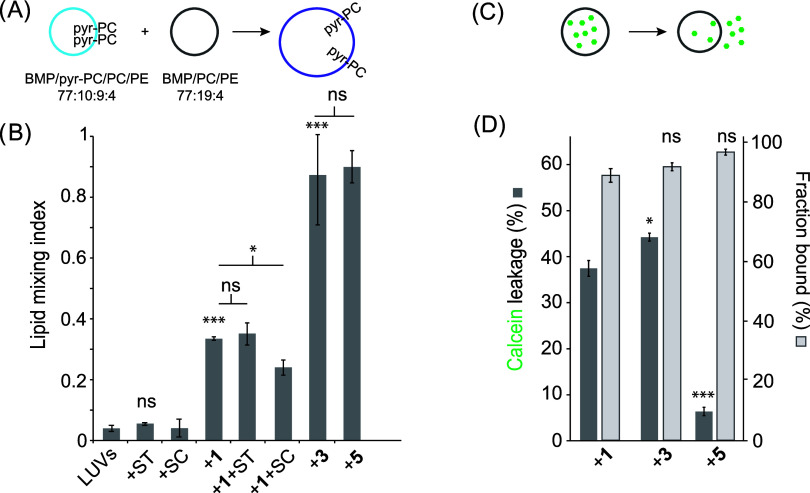
Conjugation of dfTAT to SnoopCatcher prevents the leakage of calcein-loaded LUVs with late endosome lipid composition. (A) Graphic depicting the pyrene excimer dilution assay utilized to monitor lipid mixing mediated by **1**, **3**, or **5**. LUVs doped with 10% pyrene-PC (liposomes colored cyan) were mixed with equivalent LUVs lacking pyrene-PC (liposomes colored gray) at a 1:1 ratio. (B) Quantification of lipid mixing mediated by **1**, **3**, or **5**, using a pyrene excimer dilution assay. Membrane fusion is reported as a lipid mixing index where **1** is the normalized monomer-to-excimer ratio obtained for LUVs prepared with 5% pyrene-PC. ST, SC, **1**+ST, and **1**+SC were used as controls. The *x*-axis represents LUVs treated with the indicated reagents at a peptide:lipid ratio of 1:50. (C) Graphic depicting the experiment used to assess the ability of reagents (**1**, **3**, and **5**) to cause endosomal leakage. **1**, **3**, and **5** were mixed with calcein (depicted in green) loaded LE LUVs (liposomes colored in gray). (D) Quantification of the liposomal leakage activity of **1**, **3**, and **5**, as monitored by the release of calcein. 100% leakage corresponds to the amount of calcein released in the presence of the detergent Triton X. The fraction of compound bound to liposomes, as quantified by measuring the fluorescence signal of TMR from the LUV pellet formed after low-speed centrifugation, is also provided. The data shown represent the averages corresponding to standard deviations of technical triplicates. *p ≤ 0.05, **p ≤ 0.01 ***p ≤ 0.001. Ns is not significant, or *p* > 0.05.

Based on the two-step leaky fusion model established before, the first bilayer activity tested herein was that of lipid mixing/fusion. For this assay, LUVs were doped with pyrene-PC (10%) and mixed with LUV without pyrene-PC (Figure 6A). Fusion between LUVs and lipid mixing leads to a dilution of PC-pyrene in the bilayer and a resulting increase in the ratio of pyrene monomer fluorescence (Ex = 340 nm, Em = 378 nm) to pyrene excimers (Ex = 340 nm, Em = 470 nm) (Figure 6A). For these experiments, **1**, **3**, **5**, ST, and SC were added to LUVs (1:1 doped/not doped, 500 μM total lipid) at a 1:50 construct:lipid ratio. The pyrene fluorescence was recorded and reported as a lipid mixing index, where an index of 0 corresponds to the fluorescence of 10%-dopped LUVs by themselves and an index of 1 corresponds to the fluorescence of 5%-dopped LUVs. Both ST and SC had little to no effect on lipid mixing (Figure 6B). Construct 1 was fusogenic, as previously reported.^17^ SC, co-incubated in trans, inhibited **1** only moderately. Notably, **3** and **5** were also fusogenic, displaying an apparent activity greater than that of peptide **1** despite sharing the same dfTAT moiety. This suggests a direct or indirect contribution of ST or SC in fusion. In this context, it is worth noting that the introduction of hydrophobic residues to the dfTAT structure can amplify its membrane activity.^24^ In light of this, ST and SC might impart additional hydrophobicity to **1**, thereby augmenting interactions with lipids and promoting bilayer fusion. Crucially, these findings suggest that the addition of payloads to peptide **1** does not inherently dampen the fusion activity of the peptide.

To assess the leakage activity of the constructs, **1**, **3**, and **5** were mixed with LUVs loaded with the fluorophore calcein (Figure 6C). Leakage of calcein from LUVs was quantified by monitoring free calcein present in solution after the addition of the constructs and separation by gel filtration. This assay was performed at a construct:lipid ratio of 1:50. Under these conditions, **1** and **3** caused calcein leakage (100% leakage is established by inducing bilayer disruption with triton X) (Figure 6D). In contrast, the leakage induced by **5** was 7-fold reduced compared to **3** (similar results were obtained with **4**, not shown). The fraction of constructs bound to LUVs was approximately 100% in all samples, indicating that differences observed in leakage are unlikely to be caused by differences in affinity for the lipid bilayers. This implies that the structural or physicochemical properties of SC might interfere with the mechanism through which peptide **1** induces membrane leakage. In contrast, the attachment of ST is nondisruptive to the leakage activity of **1**. When considering the combined results of these in vitro assays, we conclude that ST conjugation does not inhibit interactions with lipids and the overall leaky fusion process. In contrast, while SC conjugation does not deter the binding or fusion step, it appears to exert a specific inhibitory effect on the subsequent leakage phase.

## Discussion

This study addresses an important gap in the current understanding of cell-penetrating peptides (CPPs), specifically how the conjugation of a payload to a CPP impacts its endosomal escape. Since every new payload protein may be unique in structure and properties, establishing general rules for payload compatibility with high predictability is challenging. For instance, a negatively charged protein payload is likely to interact intramolecularly with its polycationic CPP fusion tag.^11^ Hence, some proteins may bind and alter their CPP carriers by direct interactions. To avoid this scenario, we aimed to probe conjugates where the cargos would be relatively inert toward their CPP tag. We opted for ST/SC, a protein with an isoelectric point and a surface charge that did not indicate a high likelihood of electrostatic interactions with its CPP tag. Our results confirmed that CPP and SC do not have a detectable affinity for each other. Notably, ST/SC also allowed the formation of a CPP-protein conjugate of approximately 37 kDa, a molecular weight reasonably close to the mean molecular weight of eukaryotic proteins (∼48 kDa).^35^ Hence, we envisioned ST/SC as a representative model for protein payloads with average isoelectric points and sizes. Using a modified version of the HIV-TAT peptide known as dfTAT as a model system, we therefore explored how different ST/SC payloads impact the cell-penetrating activity of this CPP. Overall, our results indicate that attachment of ST is permissible but that SC impairs dfTAT’s functionality. In this context, we propose a model where the cargo’s size and bulk limit the cell delivery activity of the CPP, as described below.

CPPs like TAT have been reported to enter cells by two possible routes, direct plasma membrane translocation and endocytosis followed by endosomal escape.^36−39^ In principle, these pathways are not mutually exclusive and may coexist under certain conditions. Notably, direct membrane plasma membrane has been observed at high peptide concentrations (10 μM or above), and, in our experience, in cells susceptible to membrane oxidation.^39,40^ Direct membrane translocation is likely disrupted by CPP attachment to large protein cargos.^40^ Our experimental data, under the conditions employed (5 μM or less for **1**, **2**, **3**), strongly suggest that the primary route adopted by the different dfTAT constructs is endocytic uptake, as evidenced by the inhibition observed in the presence of sodium azide. This intracellular trafficking pathway appeared consistent for all dfTAT variants, irrespective of payload conjugation. Specifically, all constructs accumulate in endosomal compartments. The constructs **4** and **5** remain trapped within the endocytic pathway, regardless of the incubation concentration used. In contrast, **1**, **2**, and **3** escape from the endosomes and penetrate the cytoplasm efficiently when incubated above a threshold concentration of 1 to 2 μM. The impaired endosomal escape exhibited by constructs **4** and **5** points toward two critical barriers to effective cell penetration. First, the uptake of these constructs by cells is markedly lower. Second, the attachment of the SnoopCatcher protein appears to compromise dfTAT’s ability to induce membrane leakage, a crucial aspect of endosomal escape. This dual impediment results in insufficient accumulation of the CPP within endosomes, and a reduction in the membrane activity necessary for endosomal escape.

It remains unclear why the attachment of a payload leads to a reduced level of endocytic uptake. When in trans, SC does not inhibit the ability of dfTAT to enter cells. It suggests that SC does not compete with dfTAT for binding with cell components that control its endocytosis. On the cell surface, polycationic CPPs like dfTAT interact with heparan sulfate proteoglycans such as syndecans and glypicans.^41^ Notably, it has been suggested that CPP-induced clustering of syndecans enhances endocytosis.^42,43^ Hence, one possibility is that one or two SC domains attached to dfTAT contribute steric hindrance that reduces clustering. This hypothesis, however, requires further validation.

The low propensity for endocytosis of the SC conjugates prevented us from precisely quantifying their endosomal escape activity, i.e., at endosomal concentrations equivalent to those achieved with **1** or **3**. In particular, if its endocytosis was to be improved, then **5** may be capable of escaping from endosomes. Nonetheless, *in vitro* assays indicate that this would likely not be the case because the conjugate has poor bilayer leakage activity. We have previously reported that contact between liposomes was necessary for the leakage mediated by dfTAT or other multivalent TAT analogues.^33^ In particular, polycationic CPPs bind the surface of anionic BMP-rich bilayers and bring the bilayers of liposomes in very close proximity.^44^ This phenomenon likely drives the process of both fusion and leakage and hence a description of leaky fusion. The CPP-mediated leaky fusion is dependent on the unique biophysical properties of BMP (bilayers containing phosphatidylglycerol, a regio isomer of BMP with the same charge but different fatty acid attachment positions, undergo fusion but not leakage).^33,34^ It is notable that **5** promotes lipid bilayer fusion. It is therefore capable of bringing bilayers into contact. However, while this step is necessary for leakage, it is not sufficient as **5** does not yield substantial release of calcein from the lumen of liposomes. Here again, the bulk provided by the SC moiety may be causing steric hindrance that blocks the leakage process. In particular, if one envisions structures such as transient pores potentially formed by the CPP, one can speculate that ST is small enough to allow the transit of a probe like AF488-H1 but that SC may block such pores. A study focused on TAT has proposed that the CPP generates saddle-splay membrane curvature and cross membranes through an induced pore.^45^ Notably, the attachment of a polylactide nanoparticle of 30 nm diameter blocks this process. Finally, it should be noted that TAT has successfully delivered large protein cargos into cells via apparent endosomal escape. An example reproduced in many laboratories is that of TAT-Cre recombinase.^46^ In our experience, delivery of TAT-Cre into cells requires exposure times of 16 to 24h, as opposed to the 1h used herein with dfTAT.^21^ It is therefore possible that multiple mechanisms of endosomal escape exist: some slow, some fast, and some compatible with large payloads. A CPP may therefore be intrinsically capable of modulating endosomal membranes in several ways, on different time scales and with variable efficiency. In turn, the payload may dictate which escape pathway is selected.

To fully harness the high endosomal escape activity of dfTAT and expand its utility in the future, it may be helpful to conjugate dfTAT to its payload via a linker cleaved inside endosomes. Using such an approach, dfTAT could be restored in an optimal form for endosomal leakage. Cleavable linkers that respond to the endosomal pH or to endosomal proteases have been tested before, albeit with the low escape activity TAT.^47−49^ Consistent with our results, these linkers seem to procure improvements in endosomal escape. We have yet to pursue this approach because the problem of low endocytic uptake must be solved first. For this, developing encapsulation devices that are endocytosed efficiently, regardless of the payload that they carry, would be useful. In the future, we intend to develop such capsules and combine them with dfTAT to achieve high delivery efficiencies independent of payloads.

In conclusion, our study provides crucial insights into the impact of the payload size and bulk on the cell penetration efficacy of CPPs. These findings underscore the need for careful consideration of the CPP–payload relationship in the design of effective delivery systems and pave the way for future innovations in CPP-based delivery.

## Materials and Methods

### Cell Culture

MDA-MB-213 (ATCC HTB-26) cells were cultured in Dulbecco’s minimum essential medium (DMEM) (Fisher) supplemented with 10% fetal bovine serum (FBS) (Fisher). The cells were grown in a 37 °C incubator with 5% CO_2_. For delivery and microscopy experiments, DMEM/10%FBS was exchanged with Leibovitz’s L-15 serum-free media.

### Solid-Phase Peptide Synthesis of dfTAT (**1**), dfTAT-ST (**2**), and dfTAT-dST-dSC (**3**)

Previously published protocols that describe the Fmoc-based synthesis of **1** were applied to synthesize **2** and **3**.^15,50^ First, TAT (CKRKKRRQRRRG) and TAT-ST (CKRKKRRQRRRG-KLGDIEFIKVNK) were synthesized on a rink amide MBHA resin (500 mg scale; 0.51 mmol/g loading). To site-specifically label TAT and TAT-ST scaffolds with a fluorophore, an Fmoc-Lys(Mtt)–OH (labile in 1% TFA) was coupled to the N-terminus of the resin-bound peptides. Mtt was removed with a solution of 1% TFA/1%TIS in dichloromethane (DCM). The reaction proceeded for a total of 90 min, with beads being washed with DCM, *N*,*N*-dimethylformamide (DMF), and methanol and incubated with fresh cleavage solution every 5 min.^51^ The fluorophore 5(6)-carboxytetramethylrhodamine was coupled to the deprotected ε-amine of the lysine side chain using standard HOBt coupling. The final Fmoc group on the N-terminus was removed by 20% piperidine in DMF, with successive 5 and 15 min deprotection with fresh piperidine solution. The peptides were cleaved using TIS/EDT/H_2_O/TFA 2.5/2.5/2.5/92.5. The cleavage reaction was carried out for 2 h at room temperature under agitation. Crude peptides were purified by reversed-phase HPLC using 20–40% gradients of acetonitrile in 0.1% TFA/Water. Fractions were collected and lyophilized. Dried samples were resuspended in 0.2 μM filter-sterilized H_2_O. The fTAT and fTAT-ST peptides were added to a 1:1 ratio in PBS (NaCl 137 mM, KCl 2.7 mM, Na_2_HPO_4_ 10 mM, KH_2_PO_4_, pH 7.4). Disulfide bond formation and dimerization of the peptides were monitored by HPLC. The dimerized products **1** (molecular weight = 4076 Da), **2** (molecular weight = 5461 Da), and **3** (6853 Da) were purified by reversed-phase HPLC, and their mass was analyzed by using a Bruker Ultraflex Xtreme Maldi-TOF (Texas A&M Protein Chemistry Laboratory).

### SnoopCatcher Ligation Reaction with dfTAT-SnoopTag Constructs

SnoopCatcher (SC) was recombinantly expressed and purified as previously described by Howarth and co-workers.^25^ In preparation for the ligation reaction, dried powder aliquots of **2** and **3** were resuspended in TBS (20 mM Tris, 150 mM NaCl, and 7.4 pH). SC (40 μM) was reacted with **2** (10 μM) for 1 h. Similarly, SC (80 μM) was reacted with **3** (10 μM). To track the kinetics and completion of the reaction, samples were collected every 10 min, immediately added to SDS loading buffer, and then boiled for 5 min. Samples were analyzed on SDS-PAGE (16% acrylamide) and detected by in-gel fluorescence using a Typhoon fluorescence scanner and by Coomassie staining., Cation exchange chromatography was performed to purify the products **4** and **5** formed, exploiting the cationic TAT to separate TAT labeled-SC from unreacted SC. Samples were diluted in sodium phosphate buffer (0.1 mM Na_2_HPO_4_, 0.1 mM NaH_2_PO_4_, pH 7.4) and were purified on cation exchange chromatography (HiPrep Q FF 5 mL Cytiva) using a 0.05 to 1 M NaCl gradient. The samples were then dialyzed in PBS (pH 7.4) to remove the excess salt. The purity of the final products **4** and **5** were assessed by SDS-PAGE, and their mass (expected as 20235 and 36403 Da, respectively) was analyzed by a Bruker Ultraflex Xtreme Maldi-TOF (Texas A&M Protein Chemistry Laboratory).

### Live Cell Microscopy

Fluorescence and bright-field 20× images were captured using an EVOS M7000 (Thermo Fisher Scientific) microscope. The filter sets used included: red fluorescent protein (RFP; Ex = 531 ± 20 nm and Em = 593 ± 20 nm) for detecting TMR-labeled constructs, green fluorescent protein (GFP; Ex = 470 ± 11 nm and Em = 525 ± 25 nm) for detecting AF488-H1, DAPI (Ex = 357 ± 22 nm and Em = 447 ± 30 nm) for detecting Hoechst 33342 trihydrochloride trihydrate dye (Hoechst), and Cyanine 5 (Ex = 628 ± 20 nm and Em = 685 ± 20 nm) for detecting DRAQ7. was conducted on an Olympus IX70 spinning-disk confocal microscope was used for 100× imaging with the following filter cubes RFP (Ex = 560 ± 20 nm and Em = 630 ± 35 nm) for detecting dfTAT, fluorescein isothiocyanate (Ex = 488 ± 10 nm and Em = 520 ± 20 nm) for detecting AF488-H1, and DAPI (Ex = 350 ± 50 nm and Em = 460 ± 25 nm) for detecting Hoescht. For all experiments, fluorescent species were imaged individually in all channels to confirm the absence of fluorescence cross-talk between channels under the imaging conditions used.

### dfTAT Conjugate-Mediated Delivery of AF488-H1

MDA-MB-213 cells were washed three times with L-15. The cells were incubated with molecules **1**, **2**, **3**, **4**, or **5** (5 μM) and AlexaFluor 488-labeled histone 1 (1.25 μM) (AF488-H1, Thermo Fisher Scientific) for 1 h. For the titration curves, 1, 2, 3, 4, 5, or 10 μM of **1** and **3** were incubated with cells for 1 h, while **4** and **5** were incubated with 1, 2, 5, 10, and 20 μM. To remove excess peptide after incubation with cells, the cells were washed three times with L-15 supplemented with heparin (1 mg/mL; Sigma-Aldrich). After washing, the cells were incubated for 20 min with L15 containing Hoechst (Thermo Fisher Scientific) (1 μM) and Draq7 (Thermo Fisher Scientific) (1 μM) to detect nuclei and dead cells, respectively (Figure S3). For each experiment, imaging conditions (excitation time and neutral density filter setting) were kept constant for comparison among biological replicates. Overlap of AF488-H1 and Hoechst fluorescence signals were determined using the colocalization intersection Coloc I_2_ function (ratio between a selected colocalized area above a set threshold and the area of a selected object in a parent image) in Invitrogen Celleste Image Analysis Software. Thresholds for counting overlapping pixels were kept constant among images. To perform the Coloc I_2_ analysis, a raw image containing fluorescence signals attributed to AF488-H1 was merged with its corresponding image containing signals attributed to Hoechst-stained nuclei. The image containing Hoechst signals was then set as the parent image (only overlapping fluorescence pixels are considered inside nuclei). When the I_2_ analysis is performed, each Hoechst nucleus is selected. The fluorescence signal of AF488-H1 that is contained within the bounds of Hoechst-stained nuclei is then selected. The ratio of the AF488-H1 signal contained within Hoechst-stained cells is quantified for each nucleus and is reported as I_2_. In the AF488-H1 alone control, no cells exhibited an I_2_ value above 0.4. Hence, values greater than 0.4 were considered positive for delivery.

### Colocalization of H1 with **1** or **5**

Cells were washed three times with L-15 and incubated for 1 h with AF488-H1 (1.25 μM), and either molecule **1** (0.5 μM) or molecule **5** (5 μM). The cells were washed 3 times with L-15 supplemented with heparin (1 mg/mL) and treated with Hoechst in L-15 (1 μM) for 20 min. The cells were then imaged on a 100x Olympus IX70 microscope, and individual puncta from AF488-H1, **1**, and **5** were identified using the objects tool in Celleste 5.0. The overlap of each puncta was analyzed using Celleste 5.0 colocalization tool. Puncta were binned into three categories: AF488, TMR, or AF488-H1/TMR-fluorescence. The Mander’s coefficient M1 (fraction of green fluorescence that overlaps with red fluorescence) and M2 (fraction of red fluorescence that overlaps with green fluorescence) for all puncta were analyzed. Objects exhibiting an M1 of <0.2 were considered “AF488 only” puncta, and objects with an M2 of <0.2 were considered “TMR only”. Objects with an M2 of >0.6 were considered as containing both TMR and AF488. Note that endosomes move during green and red image acquisition, leading to a shift and decrease in apparent M1 and M2 and values for colocalization less than 1. The 0.6 value was determined as a threshold for colocalization on our optical system based on the co-incubation of dextran controls labeled with the green fluorophore fluorescein (green) and the red fluorophore TMR (data not shown). Puncta with M1 or M2 of values between 0.2 and 0.6, less than 10% of the total population, were labeled as unassigned because of only partial overlap.

Using the cell profiler software, a pipeline containing the functions “RescaleIntensity”, “IdentifyPrimaryObjects”, “MeasureObjectIntensity”, and “ExportToSpreadsheet” was set up to obtain the intensity of all puncta in each image. The “RescaleIntensity” function in the cell profiler was set to stretch each image to use the full intensity range of the image. Please note that the image intensity was previously rescaled to eliminate the background signal through the software SlideBook. Therefore, the full range of intensity is from zero to the maximum punctate intensity. Using the “IdentifyPrimaryObjects” function, the lower and upper bounds of the typical diameter of objects were set to 1 and 5, respectively. The threshold for recognition of pixels was adjusted to 0.025–1.0 to eliminate pixels unrepresentative of puncta. The product produced by this pipeline was cross-referenced with the original images to ensure colocalization of the data points in question. The “MeasureObjectIntensity” function takes the product from “IdentifyPrimaryObjects” and measures the intensity of each recognized product, and the “ExportToSpreadsheet” function exports these data to an Excel sheet. The number of cells was determined by counting the number of nuclei present in the image.

### Measurement of dfTAT Conjugate Uptake

Approximately 50,000 cells were plated in 96-well plates. Constructs **1**–**5** were incubated with cells for 1 h in L-15. The cells were then lysed with 100 μL of Triton X-100-based lysis buffer (1% TX-100, 2% SDS, 150 mM NaCl, 20 mM Tris, 50 mM DTT, pH 7.4). Cell lysates were measured for TMR fluorescence using an ISS fluorometer (532 nm = λ_excitation_, 580 nm = λ_emission_). Cell lysates were normalized using a Bradford assay. Experiment was performed in biological triplicates (<50,000 cells per condition).

### Inhibition of Endocytosis by NaN_3_

Cells were plated in two separate 48-well dishes (200 μL volume per well) and grown in DMEM supplemented with 10% FBS. After 24 h, the cells were placed in L-15 medium. The cells were incubated with NaN_3_ (40 mM in L-15) for 30 min. The cells were then incubated for 1 h with **1**, **3**, or **5** (5 μM), and with AF488-H1 (1.25 μM) as a control. The cells were then washed and treated with Draq7 (1 μM) for 10 min. The cells were then imaged using fluorescence microscopy (as previously described). Cell death, as detected with Draq7, did not exceed 5% for all conditions. To prepare cells for flow cytometry, the cells were washed thrice with heparin in L-15 (1 mg/mL). The cells were dissociated from the dish by removing all media and applying 50 μL trypsin (0.25% v/v in PBS) for 3 min. The cells were resuspended in 350 μL of L-15 and were kept on ice. The fluorescence signal of the TMR-TAT was detected on a flow cytometer (BD Accuri C6 model) using a standard FL2 (λ_ex_/λ_em_ = 585/640 ± 30 nm) channel. Data were acquired at a flow rate of 66 μL/min with at least 20,000 events. The fluorescence signals were processed in FlowJo v10.8.

### dfTAT and dfTAT Conjugate-Mediated Leakage of Calcein-Loaded Liposomes

Leakage of liposomes was performed as previously described.^32^ Briefly, lipid cakes were prepared to mimic late endosomal membranes by mixing the lipids BMP, PC, and PE in a ratio of 77:19:4, respectively. Lipid cakes were then resuspended in LUV purification buffer (70 mM calcein, 100 mM NaCl, and 10 mM sodium phosphate buffer, pH 7.4) to a concentration of 10 mM to form MLVs. Ten freeze–thaw cycles were performed to form multilamellar vesicles (MLVs). The MLV mixture was extruded by using a 0.1 μM filter to generate large unilamellar vesicles (LUVs) with an approximate 100 nm diameter. The size of the LUVs was confirmed by Dynamic Light Scattering (DLS, Zetasizer Nano Malvern Panalytical). Free calcein was separated from liposomes by size exclusion chromatography (Sephadex G50 Cytiva). Stock aliquots of **1**, **3**, and **5** were diluted in leakage buffer (100 mM NaCl, 10 mM sodium phosphate buffer pH 5.5) and added to LUVs in a peptide:lipid ratio of 1:50 (final peptide concentration 11 μM and final LUV concentration 550 μM, 250 μL total volume). The peptide:liposome mixture was incubated for 1 h at room temperature. Flocculated material was spun down at 4000*g*, and the supernatant was then collected (no lipid was present in the supernatant after centrifugation, as established by phosphate analysis). The supernatant was analyzed by size exclusion using Sephacryl G-50 resin (5 mL). Absorbance of calcein was detected at 485 nm with a diode array. To calculate the percent leakage, the area under the curve corresponding to free calcein in the HPLC chromatogram was compared to a 100% leakage control obtained from the addition of 1% Triton X-100 to LUVs. To calculate the fraction of constructs **1**, **3**, and **5** bound to LUVs, supernatants obtained from centrifugation were also analyzed for TMR signal. Samples were diluted in TBS containing 50 mM DTT (to reduce dfTAT) and 2% Triton X-100. After 15 min, the TMR fluorescence of the samples was analyzed using an ISS fluorimeter (532 nm = λ_excitation_, 580 nm = λ_emission_). The fluorescence signal detected in the supernatant was divided by the fluorescence signal detected if no liposomes are present, yielding unbound and LUV-bound fractions. The experiments were performed as a technical triplicate.

### Fluorescence Anisotropy

Fluorescence anisotropy of dfTAT in the presence of BSA or SC was measured as previously described.^52^ Briefly, an ISS fluorimeter was equipped with polarizers. Then the sample chamber was heated to 25 °C for 30 min. Meanwhile, the samples were prepared. BSA and SC were prepared in separate stock solutions to a concentration of 690 nM in TBS (150 mM NaCl and 20 mM Tris, pH 7.4). The fluorescence peptide dfTAT was prepared to a concentration of 1 μM in TBS. To prepare a sample for measurement, small volumes of the stock solutions of dfTAT were mixed with the stock solutions of the target protein (BSA or SC) and diluted in TBS (brought to a final volume of 100 μL in a quartz cuvette). This resulted in the final concentration of dfTAT, and the target protein (BSA or SC) being 30 nM. The sample was then incubated for 15 min in the sample chamber at 25 °C. More specifically, samples were excited with vertically polarized light (532 nM laser) with a slit width of 8 nm. Emission measurements were then made vertically (*I*_VV_) and horizontally (*I*_VH_), and then passed through a photon multiplier tube with a cutoff filter (>580 nm light allowed through the filter). The fluorescence anisotropy (*r*) was calculated using software *Vinci*. This procedure was repeated in triplicate. To construct a titration curve, dfTAT was held constant while BSA or SC was titrated in increasing concentrations. New stock solutions with increased concentrations of BSA and SC were prepared for each data point.

## Supporting Information Available

Chemical structures of constructs **1**, **2**, and **3**; flow cytometry and fluorescence microscopy analyses of cells incubated with reduced and monomeric analogues of constructs **1**, **3**, and **5**; and cell viability analysis of cells incubated with **1**, **3**, and **5** (PDF)
