# Supplementary material for: Elucidating the Impact of Payload Conjugation on the Cell-Penetrating Efficiency of the Endosomal Escape Peptide dfTAT: Implications for Future Designs for CPP-Based Delivery Systems
Source: Bioconjug Chem. 2023 Sep 29;34(10):1861–72. doi: 10.1021/acs.bioconjchem.3c00369 (PMC10644971; doi:10.1021/acs.bioconjchem.3c00369)
Supplement: Supplementary file 1 — bc3c00369_si_001.pdf [file bc3c00369_si_001.pdf]

Elucidating the Impact of Payload Conjugation on the Cell-Penetrating Efficiency of the Endosomal Escape Peptide dfTAT: Implications for Future Design of CPP-based Delivery Systems.

Joshua Diaz[1], Miles Pietsch[1], Marissa Davila[1], Gerardo Jaimes[1], Alexis Hudson[1], & Jean-Philippe Pellois[12*]

From [1]Department of Biochemistry and Biophysics, Texas A&M University, College Station, TX 77843, USA; [2]Department of Chemistry, Texas A&M University, College Station, TX 77843, USA

*To whom correspondence should be addressed

Address correspondence to: Jean-Philippe Pellois, Biochemistry and Biophysics Bldg., Room 430, 300 Olsen Blvd, College Station, TX, 77843-2128.

Fax: 979-862-4718, E-mail: pellois@tamu.edu

Orcid ID: Joshua Diaz (0000-0001-7957-6747), Jean Philippe Pellois (0000-0001-8528-4652)

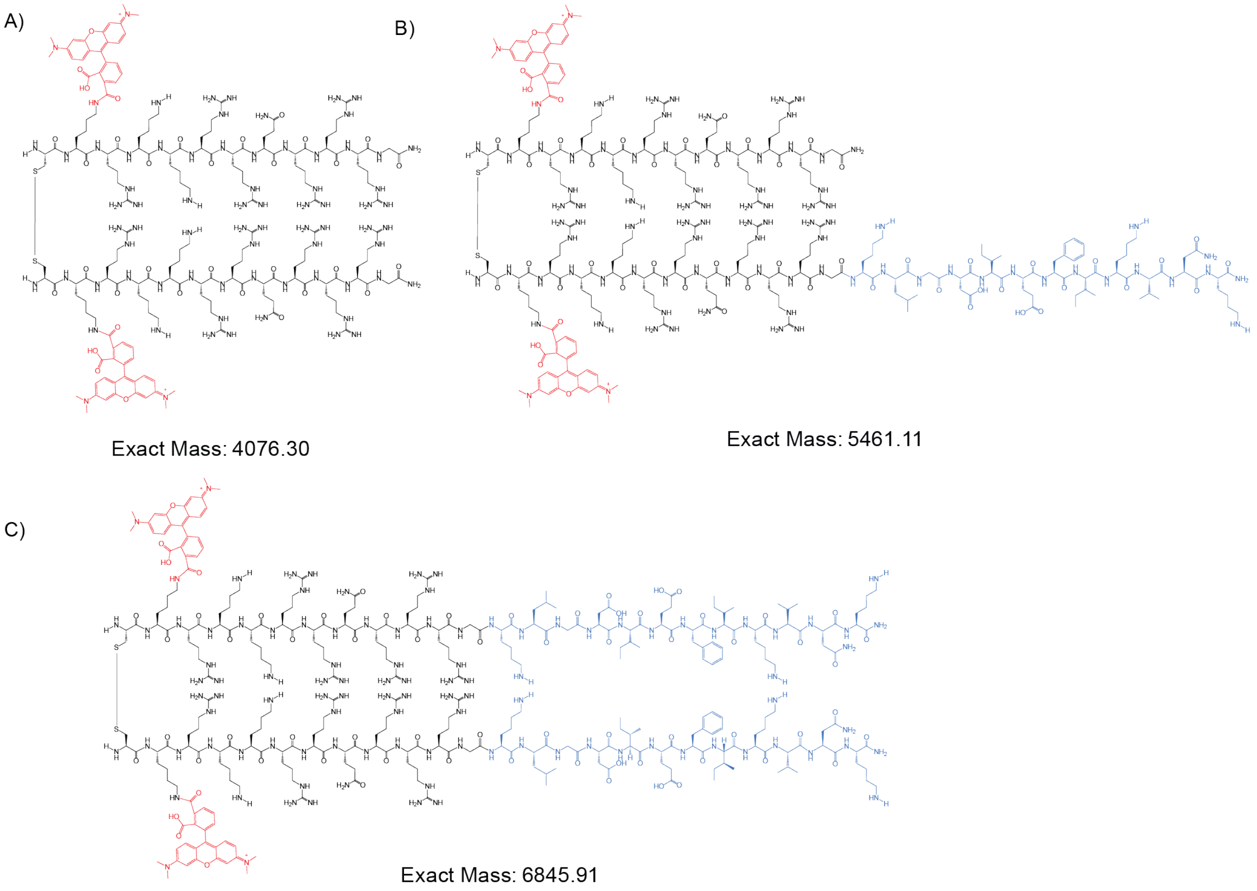

Figure S1. Structures of **1**, **2**, and **3**.

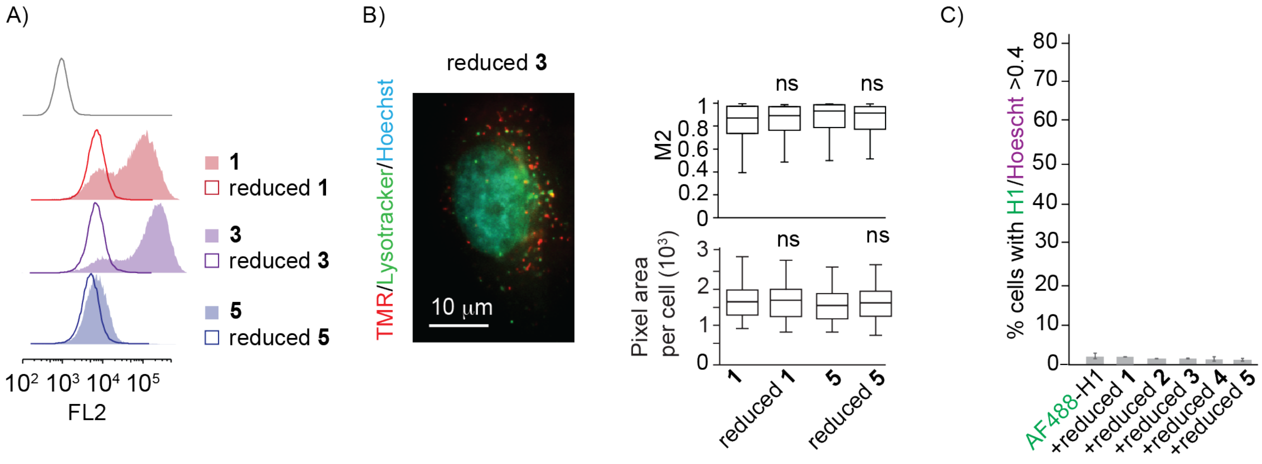

Figure S2. Analysis of reduced constructs **1**, **3**, and **5**. The constructs were reduced to their monomeric form by treatment with TCEP (50 μM, 30 min). Reduction was confirmed by HPLC and SDS-PAGE. A) Flow cytometry analysis of **1**, **3**, **5** (5 μM) before and after reduction. B) Representative microscopy image of cells incubated with reduced **3** and with Lysotracker green as in Figure 4. Colocalization between Lysotracker green and **1** or **5**, before and after reduction. The Mander's colocalization coefficient M2 (fraction of red signal that overlaps with green) is provided as a box and whisker plot. The total pixel area per cell that corresponds to fluorescent puncta and the median intensity of these pixels are shown. At least 500 cells from were analyzed of per replicate, with 3 biological replicates performed for each incubation conditions. C) Quantification of the percentage of cells displaying an H1/Hoechst colocalization greater than 0.4 after incubation between AF488-H1 and **1-5**, before and after reduction.

Overall, we conclude that the monomeric forms of **1-5** accumulate in endosomes following endocytic uptake. However, they lack the ability mediate endosomal escape and do not promote AF488-H1 release from endosomes. This is consistent with a previous report establishing that the monomeric form of **1**(dfTAT), fTAT, does not mediate detectable endosomal escape (Erazo-Oliveras, A., Najjar, K., Dayani, L., Wang, T. Y., Johnson, G. A., and Pellois, J. P. (2014) Protein delivery into live cells by incubation with an endosomolytic agent, *Nat Methods 11*, 861-867.) This is the case under conditions where fTAT is present in the lumen of endosomes at concentrations higher than that of dfTAT. Consistent with these results, fTAT does not cause leakage of liposomes mimicking the lipid composition of late endosomes, while dfTAT does (Erazo-Oliveras, A., Najjar, K., Truong, D., Wang, T. Y., Brock, D. J., Prater, A. R., and Pellois, J. P. (2016) The Late Endosome and Its Lipid BMP Act as Gateways for Efficient Cytosolic Access of the Delivery Agent dfTAT and Its Macromolecular Cargos, *Cell Chem Biol 23*, 598-607).

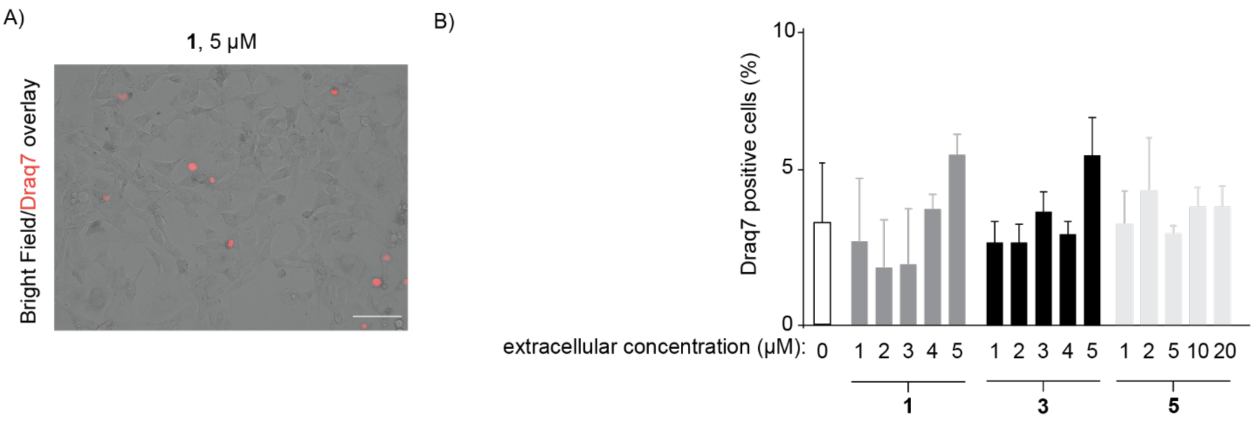

Figure S3. SnoopTag and SnoopCatcher dfTAT conjugates cause minimal toxicity in MDA-MB-231 cells. A) Microscopy images of cells incubated with 5 μM pf 1 for 1 h. Cells are then washed and stained with Draq7 (scale bar = 100 μm). The image is an overlay of a bright field image and Draq7 fluorescence image. B) Toxicity of 1,3, and 5 as detected by counting Draq7 positive cells. Cells were treated with constructs at represented extracellular concentrations for 1h. After treatment, cells were stained with Hoechst (1 μM) and Draq7 (1 μM) for 20 minutes before imaging (> 300 cells, biological duplicate). The number of Hoechst-stained nuclei is used to determine the total number of cells, while the Draq7-stained nuclei are counted as dead cells. The data represent the average and corresponding standard deviations of biological triplicates, 1500 cells being analyzed per condition.
